# Immunogenicity of Externally Activated Nanoparticles for Cancer Therapy

**DOI:** 10.3390/cancers12123559

**Published:** 2020-11-28

**Authors:** Onur Sahin, Ashokkumar Meiyazhagan, Pulickel M. Ajayan, Sunil Krishnan

**Affiliations:** 1 Department of Materials Science & NanoEngineering, Rice University, Houston, TX 77005, USA; os8@rice.edu (O.S.); ajayan@rice.edu (P.M.A.); 2 Department of Radiation Oncology, Mayo Clinic Florida, 4500 San Pablo Road S, Mayo 1N, Jacksonville, FL 32224, USA

**Keywords:** radiosensitizing nanoparticles, X-ray, rare-earth elements, photothermal therapy, photodynamic therapy, magnetic hyperthermia

## Abstract

**Simple Summary:**

Recent advances in treating cancer via stimulating an anti-tumor immune system response have resulted in extraordinary results for lymphomas and leukemias; however these therapies have not performed well in solid tumors. External beam therapies, such as radiotherapy, hyperthermia, and photodynamic therapy, that are clinically used for solid tumors are now being explored in combination with nanoparticle systems to stimulate a long-term anti-tumor immune system response. In this review, we detail the novel nanoparticle complexes that are being researched to activate an anti-tumor immune response in combination with external beam therapy in both the preclinical and clinical settings.

**Abstract:**

Nanoparticles activated by external beams, such as ionizing radiation, laser light, or magnetic fields, have attracted significant research interest as a possible modality for treating solid tumors. From producing hyperthermic conditions to generating reactive oxygen species, a wide range of externally activated mechanisms have been explored for producing cytotoxicity within tumors with high spatiotemporal control. To further improve tumoricidal effects, recent trends in the literature have focused on stimulating the immune system through externally activated treatment strategies that result in immunogenic cell death. By releasing inflammatory compounds known to initiate an immune response, treatment methods can take advantage of immune system pathways for a durable and robust systemic anti-tumor response. In this review, we discuss recent advancements in radiosensitizing and hyperthermic nanoparticles that have been tuned for promoting immunogenic cell death. Our review covers both preclinical and clinical results, as well as an overview of possible future work.

## 1. Introduction

In recent years, immunotherapies utilizing chimeric antigen receptor (CAR) T cells and immune checkpoint blockades have shown remarkable success in treating cancer. While these groundbreaking advances have performed well in treating blood cancers such as lymphomas and leukemias, there has been limited success in translating the triumph of CAR T cell therapy and immune checkpoint blockade to solid tumors and immunologically cold tumors [1]. To overcome the current limitations in treating solid tumors via immunotherapies, methods that stimulate an antitumor immune response via immunogenic cell death (ICD) have garnered significant research attention. External beam therapies, such as radiotherapy, photodynamic therapy, and hyperthermia, have been shown to cause ICD, which can stimulate antitumor immune system effects that inhibit both primary tumor growth and secondary metastases and confer long-term immunity against re-challenge [2]. These externally activated systems are often mediated via nanoparticle materials that can both enhance immunogenic effects by increasing the efficacy of these therapies as well as act as drug-delivery vehicles for the local release of immunotherapeutics. In this review, we explore examples of nanotechnology being used in external beam therapies to stimulate damage-associated molecular patterns (DAMPs), pro-inflammatory cytokines, and tumor-related antigens.

DAMPs can be broadly grouped into two categories: those expressed on the cell surface, and those that are released from the cell. The most commonly studied cell surface DAMP is calreticulin, a chaperone protein normally found in the endoplasmic reticulum (ER) of cells. In the early stages of ICD, calreticulin translocates from the ER to the extracellular surface of the cell membrane, where it provides an “eat me” signal to phagocytic cells. Interestingly, this pro-phagocytic signal can be counteracted by the expression of CD47 on tumor cells, which is found in a variety of solid tumors [3]. Similarly, heat shock protein 70 (HSP70), another chaperone protein normally found intracellularly, can translocate to the outer leaflet of the plasma membrane during ICD, where it can mediate CD8^+^ T cell responses, promote natural killer (NK) cell activity, and stimulate dendritic cell (DC) maturation [4]. 

ICD is also associated with a variety of compounds that stimulate an immune response when released extracellularly. High mobility group box 1 (HMGB1), a nuclear protein, is released due to the breakdown of both the nuclear membrane and cell membrane during ICD. Once HMGB1 is in the extracellular milieu, it can mediate an immune response via binding to toll-like receptors (TLRs) such as TLR4 and TLR2. Extracellular ATP has also been studied as a marker for ICD. Once released, extracellular ATP leads to the activation of the NLRP3 inflammasome, resulting in the secretion of interleukin-1β (IL-1β) and IL-18, which are pro-inflammatory cytokines that can recruit both innate and adaptive immune system cells [5]. During ICD, mitochondrial contents, such as mitochondrial DNA (mtDNA), can also be released, stimulating an immune response. Specifically, mtDNA, containing cytosine-guanine (CpG) sequences that are unmethylated, can activate neutrophils via TLR9 pathways [6]. In addition to mitochondrial contents, cytosolic material, such as uric acid, can also lead to an immune response via NLRP3 inflammasome activation [7].

Cytokines are cellular signaling compounds essential for mediating an immune response. Three important categories of cytokines that are involved in ICD pathways include interferons (IFNs), tumor necrosis factors (TNFs), and interleukins (ILs). IFNs, which are signaling cytokines named for their viral interference capability, can be further subdivided into three types: type I, type II, and type III. One of the most well-studied IFNs related to tumor biology is IFN-γ, which is the only member of the type II group and is produced by both NK and CD8 T cells to stimulate antitumor activity; however, there is mounting evidence that IFN γ can also activate immune suppression pathways [8]. Type I IFNs, such as IFN-α and IFN-β, have also garnered significant attention for activating NK cells and DCs [9]. Similarly, immunogenic cell death can involve TNF-α secretion via macrophages, which can lead to both tumor promotion as well as an antitumor activity, depending on the context [10]. Finally, interleukins, which are signaling compounds released by leukocytes, play a pivotal role in controlling immune responses. Some important interleukins relevant to ICD include IL-1β, IL-2, IL-6, and IL-12, which, when released by both innate and adaptive immune cells, can promote antitumor inflammatory response [11].

To promote ICD, externally activated cancer treatment systems have been extensively explored. These systems include hyperthermia, photodynamic therapy, and radiotherapy and can be mediated via nanoparticles to induce ICD. In this work, we reviewed trends in the current literature analyzing the immunogenicity of these externally activated cancer treatments.

## 2. External Beam Systems

### 2.1. Hyperthermia

Hyperthermia is a treatment modality where either near-infrared (NIR) laser light or alternating magnetic fields can be used to locally increase the temperature of tumor tissue to 35–40 °C. Nanoparticles are used to mediate the conversion of the energy in these fields to heat [12,13], which can result in immunogenic cytotoxicity due to the stimulation of HSP pathways as well as the expression of DAMPs and the increase in cytokine production [14].

### 2.2. Photothermal Therapy

Photothermal therapy (PTT) typically utilizes NIR lasers that penetrate deep through normal tissues (and tumors) via the so-called biological optical window where there is minimal absorption by native chromophores like oxyhemoglobin, deoxyhemoglobin, water, and melanin. Surface plasmons on the outer layer or shells of nanoparticles can be tuned to the wavelength of incident NIR to create resonant wavelengths that efficiently convert light to heat, i.e., photothermal activation. In recent years, researchers have focused on the fabrication of PTT nanoparticles loaded with immunotherapies for performing a combination of hyperthermia and immunotherapy to stimulate an antitumor immune response.

**Figure 1:**
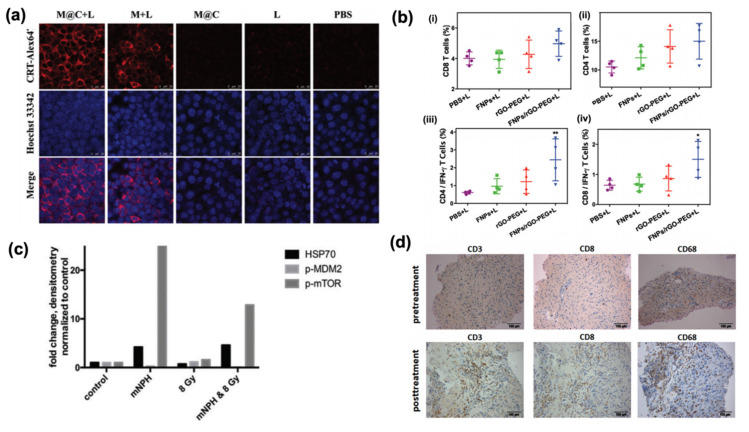
Immunogenic effects of hyperthermia treatment. (**a**) Analysis of the expression of surface-exposed calreticulin after treating 4T1 cells with melanin nanoparticles (reproduced with permission from *Chemical Communications*, published by Royal Society of Chemistry, 2020) [15]; (**b**) T cell subpopulations found in spleens of 4T1 tumor-bearing mice treated with reduced-graphene oxide hybridized iron oxide nanoparticles (reproduced with permission from *Journal of Materials Chemistry B*, published by Royal Society of Chemistry, 2020) [16]; (**c**) Expression of immunogenic factors evaluated after combined hyperthermia with magnetic nanoparticles and radiotherapy in B16 murine melanoma cells [18]; (**d**) Paraffin-embedded tissue sections stained for T cell subpopulations taken from patients treated with hyperthermia using superparamagnetic iron oxide nanoparticles [19].

For example, Li et al. [15] fabricated a PTT agent via extracting melanin from the ink sac of cuttlefish (Figure 1a). Their melanin nanoparticles, which exhibit strong photothermal activation with NIR light, were then coated with cancer cell membranes to aid in trafficking to the tumor site and loaded with indoleamine 2,3-dioxygenase (IDO) inhibitors to overcome the immunosuppressive effects of the tumor microenvironment. Interestingly, mice in the treatment group had higher levels of IL-6 and IL-12, as well as more calreticulin translocation to the cell surface, than those in the control group. In a similar study, Wang et al. [16] synthesized reduced graphene oxide hybridized to iron oxide nanoparticles (Figure 1b). While the graphene oxide acted as the PTT agent, the iron oxide nanoparticles allowed for tracking via magnetic resonance imaging. The hybridized particles were also PEGylated to improve circulation. After treatment, serum concentrations of IL-6 and IL-12 both increased, as did activation of CD8 and CD4 T cells in mouse spleens. The expression of DAMPs, such as calreticulin translocation and extracellular HMGB1, was also observed in vitro. Additionally, Zhang et al. [17] fabricated a nanocarrier composed of an organic PTT agent, IR820, linked to the IDO inhibitor, 1-methyl-tryptophan. The linked compounds then self-assembled into micelles for efficient delivery to the tumor. In vitro experiments conducted with B16F10 melanoma cells showed an increase in calreticulin and HSP70 expression. Interestingly, when combined with αPDL1 therapy, their nanoparticle resulted in increased tumor cytotoxicity, lymphocyte trafficking to the tumor, and CD4 and CD8 T cell activation.

PTT can also be combined with additional processes, such as reactive oxygen species generation, to improve the cytotoxicity of NIR light. Yan et al. [20] synthesized a dual therapy compound that can perform both PTT and photodynamic therapy (PDT). They constructed a core-shell nanoparticle with polydopamine as a photothermal core surrounded by a NaGdF_4_: Yb/Er shell modified with chlorin e6. Under NIR irradiation, the polydopamine core had a PTT effect, while the shell upconverted the NIR light to green light, which was absorbed by chlorin e6 to generate reactive oxygen species. To evaluate ICD, the group measured the DC maturation in tumor-draining lymph nodes and CD8 T cell activation in the spleen via murine in vivo models. After therapy, both DC maturation and CD8 T cell activation increased compared to controls. Interestingly, when combined with PD-L1 immune checkpoint blockade, they also found an increase in various cytokines in the serum such as IL-6 and TNF-α. Additionally, Kang et al. [21] synthesized a combined PTT/PDT complex by loading chlorin e6 onto gold nanorods coated with mouse serum protein corona. Under NIR irradiation, the gold nanocages induced PTT, while the chlorin e6 generated reactive oxygen species for PDT. The resulting ICD resulted in the activation of DCs and macrophages in an in vitro model. Li et al. [22] constructed a PTT agent to act as an amplifier of ICD. A double-layered copper hydroxide nanostructure was used for PTT with FeOOH nanodots embedded in the layers to generate reactive oxygen species and an HSP90 inhibitor loaded in between the layers to prevent HSPs from preventing thermal damage. In both in vitro and in vivo models, their nanostructure led to calreticulin translocation to the cell surface when excited by NIR lasers and increased the number of activated CD8 T cells in both the tumor and spleen in mouse models after treatment.

PTT agents have also been coupled with traditional anti-cancer therapies. Wen et al. [23] synthesized a PTT agent loaded with chemotherapy to trigger ICD. Doxorubicin and palladium were loaded into a triglyceride monostearate carrier, which could be degraded by matrix metalloproteinase 2 (MMP2), an enzyme that is overexpressed in the tumor microenvironment. Once MMP2 cleaves the carrier apart, the chemotherapeutic, doxorubicin, and the PTT agent, palladium nanoparticles, localize within the tumor, resulting in ICD. The in vitro immunogenic effects of this combination therapy were quantified by measuring cell surface calreticulin, HMGB1 expression, and extracellular ATP, which were all increased in the treatment group. In vivo studies were also performed, showing larger amounts of ATP secretion and calreticulin translocation in tumor tissue from the treatment group as well as higher concentrations of TNF-α and IFN-γ in serum taken from treated mice. Similarly, Wang et al. [24] constructed a phospholipid nanocarrier loaded with a PTT agent, named DiR, and either mertansine or vadimezan as a co-therapeutic. Interestingly, a greater immunogenic response was observed when vadimezan was loaded into the nanocarrier, as seen by an increase in TNF-α, IFN-γ, IL-12, and DC maturation, which the researchers attribute to the cytotoxicity of mertansine against DCs.

Gold is another popular material utilized extensively in the PTT literature. Ma et al. [25] prepared fluidic liposomes embedded with gold nanoparticles in the second NIR biological window (1000 nm–1700 nm). Their complex increased DAMP expression both in vitro and in vivo as measured via calreticulin and HMGB1 release and increased serum levels of IL-6, IFN-γ, and TNF-α. Additionally, an increase in CD8 T cell and DC activity was also observed in both mouse spleens and tumors. Chang et al. [26] synthesized a Cu_2_MoS_4_/Au heterostructure as a theranostic agent and showed that it could also stimulate an antitumor immune response. Under NIR irradiation, tumor-bearing mice had higher activations of both CD4 and CD8 T cells in mouse spleens in treatment groups as well as an increase in IL-6, IL-12, and TNF-α in mouse serum.

2D nanomaterials have also been used for immunogenic PTT as shown by Zhao et al. [27], who synthesized black phosphorus 2D nanosheets with high PTT efficiency that caused ICD. This was characterized by an increase in TNF-α and IL-6 in serum as well as the expression of necrotic proteins such as RIP1 and RIP3.

### 2.3. Magnetic Field

Magnetic hyperthermia is a cancer treatment strategy that utilizes magnetic nanoparticles, typically ferrites, activated by alternating magnetic fields [13,28]. Under these alternating fields, magnetic nanoparticles exhibit hysteresis, which produces localized heat, and thereby results in tumor cytotoxicity.

In an in vitro study, Duval et al. [18] studied the immunogenicity of magnetic hyperthermia by itself as well as when combined with radiotherapy using a B16 murine melanoma model. While cells exposed to either a hyperthermic dose of 43 °C for 30 min or an 8 Gy radiotherapy dose exhibited an increase in HSP70 and toll-like receptor pathways, in the combination therapy, cells also had elevated expression of chemokines such as CXCR3, CXCL10, and CXCL11 (Figure 1c).

To perform in vivo studies, Wang et al. [29] synthesized a Janus nano bullet capable of both magnetic hyperthermia and photodynamic therapy. Magnetic mesoporous organosilica nanoparticles were fabricated then loaded with the photosensitizer chlorine e6. This construct was then coated by a cancer cell membrane vesicle to promote transportation to the tumor. After entering tumor cells, reductive conditions within the cell degrade the construct, releasing the photosensitizer and the magnetic nanoparticle. In an in vitro study, their particles elicited an immunogenic response in an MCF7 breast cancer cell line, as measured via the increase in calreticulin and HMGB1 expression as well as an increase in DC maturation when co-cultured. In vivo studies showed an increase in serum HMBG1, TNF-α, IFN-γ, and IL-6 as well as an increase in the ratio of CD8/CD4-activated T cells in tumors from the treatment group. When combined with an anti-CTLA-4 immune checkpoint inhibitor, regulatory T cells in the tumor microenvironment also decreased compared to controls. In a similar study, Liu et al. [30] synthesized a ferrimagnetic nanoring structure capable of magnetic hyperthermia, which, when combined with anti-PDL1 therapy, resulted in significant tumor cytotoxicity as well as reduced incidence of lung metastases and infiltration of activated T cells into the tumor.

Though iron oxide materials are typically used, Chao et al. [31] synthesized a pure iron core nanoparticle complex, as opposed to the standard iron oxide, loaded within a liposome to prevent oxidation, which was then PEGylated. In vivo studies showed the efficacy of this particle as a magnetic hyperthermia agent. In addition to treating the primary tumor in mice, this group showed that their pure iron complex resulted in ICD, producing an abscopal effect. Additionally, when combined with anti-CTLA-4 checkpoint blockade therapy, their system resulted in an increase in both TNF-α and IFN-γ in serum.

Clinical use of immunogenic hyperthermia has also been explored. In a clinical study, Grauer et al. [19] reported the immunogenic effects of magnetic hyperthermia and radiotherapy in six patients with recurrent glioblastoma (Figure 1d). After the resection cavity was coated with iron oxide nanoparticles, each patient received six rounds of 1 hr hyperthermia followed by a 39.6 Gy dose of concurrent fractionated radiotherapy. After treatment, biopsies taken from the tumor showed an increase in necrosis as well as an increase in HSP70. Additionally, there was increased infiltration of macrophages and activated T cells. Two patients also had a long-term response, resulting in survival durations longer than 23 months without needing additional therapy. An additional clinical study was performed by Maier-Hauff et al. [32], where 59 recurrent glioblastoma patients were treated with hyperthermia mediated via iron oxide nanoparticles in combination with a reduced dose of radiotherapy, which resulted in an increased overall survival following first tumor recurrence.

## 3. Photodynamic Therapy

Photodynamic therapy (PDT) is the process whereby a photosensitizer, generally activated by a UV-vis light source, produces reactive oxygen species (ROS) in the tumor, resulting in cytotoxicity. Due to the ability of ROS to result in necrotic cell death, there has been significant interest in studying the immunogenic effects of PDT. To deliver the PDT complex and release the photosensitizer in the tumor, Yang et al. [33] synthesized a pH-responsive vesicle made from a block copolymer polyethylene glycol-b-cationic polypeptide and loaded it with a photosensitizer as well as an IDO inhibitor. In vitro studies in B16F10 murine melanoma cells showed an increase in calreticulin translocation after therapy, and in vivo studies noted an increase in TNF-α and IL-6 as well as an increase in activated T cell infiltration into primary and metastatic tumors. To further aid in delivery to the tumor location, Yu et al. [34] fabricated a nano complex using zinc phthalocyanine as a photosensitizer coated in bovine serum albumin to aid in delivery to the tumor. In an osteosarcoma mouse model, they showed that the ROS produced from their system resulted in an increase in calreticulin translocation as well as an inhibition of PD-L1 expression. Interestingly, when combined with the autophagy inhibitor, 3-Methyladenine, these results were improved.

**Figure 2:**
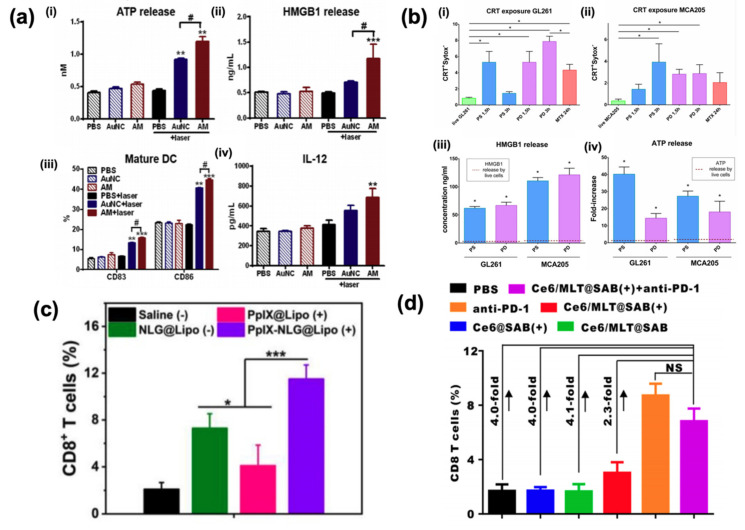
Immunogenicity of photodynamic treatments. (**a**) Evaluation of immunogenic factors in treating 4T1 cells with photodynamic therapy using gold nanocages coated in manganese dioxide, (reproduced with permission from *Biomaterials*, published by Elsevier, 2019) [36]. **, *p* < 0.01; ***, *p* < 0.001; #, Differences between two groups are statistically significant, #, *p* < 0.05; (**b**) Analysis of immunogenicity of GL261 and MCA205 cells treated with photodynamic therapy using photosen (PS) or photodithazine (PD) as a photosensitizer [37]. *, *p* < 0.006; (**c**) Infiltration of CD8^+^ T cells in 4T1 tumor-bearing mice after photodynamic treatment using PpIX and IDO inhibitor, NLG919 [40]. *, *p* < 0.05; ***, *p* < 0.001; (**d**) Analysis of in vivo immune response of photodynamic therapy co-delivered with melittin in mice bearing 4T1 tumors (reproduced with permission from *ACS Nano*, published by American Chemical Society) [42].

To overcome hypoxia, Liu et al. [35] synthesized a biomimetic system composed of photosynthetic bacteria, *Synechococcus* 7942, bound to a photosensitizer, indocyanine green coated with human serum albumin. Once the bacteria infiltrated the tumor microenvironment, laser light was used to activate both the bacteria to produce oxygen and the photosensitizer to produce ROS. In vivo studies showed a remarkable increase in ICD manifest as an increase in both NK and activated CD8 and CD4 T cells in tumor tissues as well as calreticulin translocation. In an alternative strategy to overcome hypoxia, Liang et al. [36] constructed a gold nanocage encapsulated by an Mn_2_O shell as a theranostic PDT agent (Figure 2a). When delivered to the acidic tumor microenvironment, the Mn_2_O shell degrades, providing the O_2_ necessary for PDT even in hypoxic conditions. In addition to generating ROS under NIR irradiation, the particles also produced a photoacoustic signal for imaging and could be used for MRI as well. Immunogenicity was measured via ATP and HMGB1 release, as well as DC maturation and IL-12 production in the supernatant of 4T1 cells co-cultured with DCs. In vivo studies also confirmed ICD via the increase in calreticulin translocation and the increase in T cell infiltration and DC maturation in the tumor.

In a comparative study, Turubanova et al. [37] evaluated the ICD of PDT using two types of photosensitizers, photosens and photodithazine (Figure 2b). Interestingly, while both photosensitizers resulted in calreticulin translocation and HMB1 and ATP release, photosens primarily accumulated and damaged lysosomes, whereas photodithazine accumulated in the endoplasmic reticulum and Golgi apparatus. In vitro immunogenicity was also assessed by co-culturing treated cells with DCs, which matured and produced IL-6 in co-cultures treated with both photosensitizers.

PDT agents have also been combined with immunotherapies to explore the efficacy of dual treatments. Bao et al. [38] synthesized a targeted PDT agent by conjugating an anti-CD276 antibody to an IRDye 700 photosensitizer. Due to the overexpression of the CD276 receptor in tumor vasculature, their complex can be targeted to the tumor microenvironment and then activated to produce ROS via laser light. After treatment in an in vivo model, PD-L1 expression in the tumor was increased, priming cells for PD-L1 checkpoint blockade therapy. They also showed that their PDT therapy in combination with checkpoint blockade therapy significantly reduced metastases in their murine model. In a similar study, Zhang et al. [39] fabricated chlorine e6 combined with either nanoparticle containing the Bristol–Myers Squibb small-molecule inhibitor of the PD-1/PD-L1 interaction, BMS202, or the anti-PD-L1 antibody. After injection, these nanoparticles passively accumulated in the tumor microenvironment in murine tumor models and produced ROS under laser irradiation, resulting in ICD, which was measured via the increase in DC maturation as well as activated T cell infiltration. They concluded that a nanoparticulate small-molecule inhibitor of PDL1 may be as effective as the antibody, which faces clinical translational issues with immunogenicity, high production costs, and limited tumor penetration. Additionally, Huang et al. [40] co-loaded protoporphyrin IX with an IDO inhibitor into a liposome for delivery. In a murine model, they showed that their PDT system resulted in ICD, which stimulated an immune response as measured by an increase in CD8 T cell infiltration into the tumor as well as a reduction in distant tumor burden (Figure 2c).

To test the effects of chemotherapy and PDT, Yang et al. [41] constructed a copolymer liposome loaded with doxorubicin hydrochloride to stimulate ICD and 2-(1-hexyloxyethyl)-2-devinyl pyropheophorbide-a as a photosensitizer. Once the nanocomplex enters cells, the chemotherapy and photosensitizer are released, which produced ICD under laser irradiation as measured by an increase in the translocation of calreticulin to the cell surface in vitro and an increase in DC maturation in vivo.

Compounds that can elicit an immune response have also been studied in combination with PDT. Liu et al. [42] synthesized an aluminum hydroxide nanorod coated with serum albumin and loaded it with the photosensitizer, chlorin e6, and melittin, a peptide found in honey bee venom. Once the nanorod delivered the photosensitizer and melittin to the tumor, the melittin triggered cell death via permeabilizing the cell membrane combined with the ROS activated by laser light. This dual cytotoxic approach resulted in significant ICD as measured by the increase in DC maturation and activated T cells (Figure 2d). Meng et al. [43] synthesized a PDT gelation system composed of poly(ethylene glycol) double acrylate, which polymerizes in the presence of free radicals, loaded with chlorin e6 conjugated to catalase, as a photosensitizer and imiquimod in poly(lactic-co-glycolic acid) nanoparticles as an immune system modifier. After injecting a solution containing these compounds into the tumor and irradiating with red light, the photosensitizer produces free radicals, resulting in polymerization and long-term controlled release of the therapeutic compounds. The researchers showed that not only did their therapy result in CD8 T cells in the tumor, but, when combined with CTLA-4 checkpoint blockade, the abscopal effect was also evidenced.

## 4. Radiosensitizers

Radiosensitizers, which are compounds that can sensitize tumors to make radiotherapy more effective, have received renewed interest due to their potential for stimulating an antitumor immune response.

**Figure 3:**
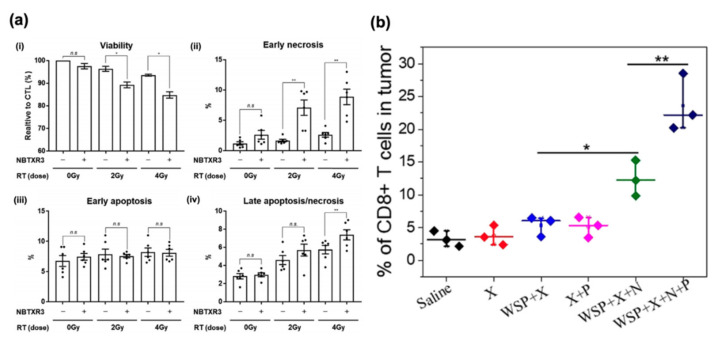
Immune response of radiosensitizers. (**a**) hafnium oxide particles increase necrotic cell death in HCT116-DUAL cancer cell line (reproduced with permission from *international journal of radiation oncology*, published by Elsevier Inc) [47]. n.s., not significant; *, *p* < 0.05; **, *p* < 0.01. (**b**) CD8+ T cell infiltration in a secondary tumor in 4T1 tumor-cell-bearing mice after treating the primary tumor with WO_2.9_-WSe_2_-PEG nanoparticles (reproduced with permission from *ACS Nano*, published by American Chemical Society) [48]. *p*-values based on Student’sttest: *, *p* < 0.05; **, *p* < 0.01.

Due to the high atomic number of hafnium, which results in greater interaction cross-sections with ionizing radiation, resulting in more energy deposition, hafnium oxide nanoparticles have been explored for immunogenic radiosensitization. Zhang et al. [44] noted that these nanoparticles, when deposited intratumorally in murine colorectal cancers, can sensitize these tumors to radiotherapy. Furthermore, these resulted in greater immune cell infiltration in the irradiated tumor as well as in distant (unirradiated) tumors. This systemic immune priming was attributed to the greater cytotoxicity of the combination therapy resulting in more of an in situ tumor vaccination effect. Galon et al. [45] tested hafnium oxide nanoparticles as radiosensitizers in patients with soft tissue sarcoma. Immunohistochemistry analysis showed an increase in CD8 and PD1 in patients who received the particles and radiotherapy versus radiotherapy alone, and gene expression analysis showed an increase in T cell activation markers as well, suggesting that the radiosensitizers could stimulate an adaptive immune response. Further clinical data of hafnium oxide nanoparticles were shown by Bonvalot et al. [46], where 179 patients were randomized between a radiotherapy + nanoparticle group and a radiotherapy only group. A complete pathological response was achieved in 16% of patients in the combination group and 8% of patients in the radiotherapy-only group. In an in vitro setting, Marill et al. [47] showed treatment with hafnium oxide nanoparticles and radiotherapy greatly increased activation of the cGAS-STING pathway due to the release of DNA into the cytoplasm from radiosensitization, which results in the secretion of type I interferons and recruitment of DC into the tumor (Figure 3a). Similarly, tungsten’s high atomic number has also received interest. Dong et al. [48] synthesized a WO_2.9_-WSe_2_ nanoparticle system that can perform both PTT and radiosensitizer, resulting in ICD at a relatively lower temperature and dose as measured by an increase in calreticulin translocation in treated 4T1 cells in vitro. In in vivo models, combination radiotherapy and PTT mediated via these particles in addition to anti-PDL1 therapy also resulted in drastic decreases in primary tumor volume, as well as an increase in CD4+ and CD8+ T cells in the tumor microenvironment and immune memory effect, which inhibited tumor growth after a secondary inoculation (Figure 3b). Bismuth, with an atomic number of 83, is also a promising candidate for inclusion in radiosensitizing compounds. Yu et al. [49] synthesized a bismuth sulfide complex conjugated to a fungal *Ganoderma lucidum* polysaccharide. Under X-ray irradiation, not only did the bismuth nanocomplex result in tumoricidal activity, but the immunogenic polysaccharide provoked a systemic immune response, resulting in the activation of CD4 and CD8 T cells as well as DCs.

To overcome hypoxic conditions, Chen et al. [50] synthesized a core-shell nanoparticle using poly(lactic-co-glycolic) acid loaded with imiquimod as the shell that encapsulates an aqueous core with catalase. After being taken up by the tumor cells, catalase decomposes H_2_O_2_ into O_2_, thereby increasing the efficacy of radiotherapy, which generates tumor antigens after treatment, allowing for imiquimod, a TLR-7 agonist, to mount an antitumor immune response, as measured by an increase in TNF-α, IFN-γ, and IL-12 in a mouse model. Additionally, the authors showed that their nanoparticle formulation, combined with CTLA-4 checkpoint blockade therapy and radiotherapy, could inhibit tumor metastases and provide long-term antitumor immunity effects when rechallenged by an additional tumor inoculation. Similarly, Zhu et al. [51] synthesized a liposome containing doxorubicin and hemoglobin to provide oxygen for overcoming the hypoxic conditions of the tumor microenvironment, resulting in increased efficacy of radiotherapy as well as ICD as measured by an increase in calreticulin translocation, HMGB1 expression, IFN-γ expression, and CD8+ T cell infiltration into the tumor in murine tumor models. A table summarizing types of nanoparticles vs triggers is shown in Table 1 which exhibits the role of nanoparticles in different systems.

## 5. Potential Limitations

Though there has been significant progress in showing that external energy sources coupled with nanoparticles can activate ICD, one possible area for improvement in these systems is the preferential delivery of these nanoparticles to tumors. Several approaches have been explored in this realm, including more stable and complete surface decorations with stealth moieties such as polyethylene glycol (PEG) [52], remodeling the tumor microenvironment [53], and/or reducing peripheral non-specific uptake in reticuloendothelial organs [13]. Achieving substantial tumor accumulation without direct intratumoral injections may allow for more widespread utilization and clinical utility.

In addition to the nanoparticle delivery concerns shared by these external beam systems, each of the modalities has individual drawbacks. For instance, one set of challenges present in hyperthermia treatment strategies involves delivering the external beam energy into deep tissues. The limited penetration depth of NIR light poses a problem for photothermal systems, and the variable power distribution of radio frequencies has similar issues in delivering accurate power distribution for localized treatments [54]. A similar penetration depth issue is present in photodynamic therapy activated by NIR light, though alternative excitation mechanisms are being explored. One such approach is to utilize an internal light source that can localize within tumors, such as radioisotopes with potent Cerenkov radiation emissions, which can then be coupled to a PTT or PDT agent [55]. Additionally, for systems involving radiotherapy, the collateral damage caused by ionizing radiation to surrounding tissue is a potential drawback.

Another possible area for future work is to broaden the scope of DAMPs studied for immunogenicity outside of the common markers such as calreticulin, HSP, and HMGB1. A few potentially interesting, yet less well studied, processes that could be explored to better understand ICD include the release of extracellular ATP as well as the release of mitochondrial ATP and intramitochondrial factors.

## 6. Conclusions

A convergence of recent evidence suggests that external energy in the form of light, X-rays, and alternating magnetic fields can, when coupled with tumor-localized nanoparticle transducers, provide efficient means of triggering ICD and thereby a robust systemic anti-tumor immune response. Due to their high spatial and temporal control, these treatment modalities offer the potential for exquisite precision in tumor cytotoxicity and immune activation. If it is indeed possible to convert an immunologically cold tumor to an immunologically hot one, this approach would have immense clinical value, especially since the resulting in situ tumor autovaccination is on-demand, extrinsically triggered, and potentially tumor-type-agnostic, making it a turn-key class solution for any tumor type.

## Figures and Tables

**Table 1:** Externally activated nanoparticle systems.

System	Trigger	Nanoparticle Complexes
Hyperthermia	NIR	Melanin [15], graphene oxide with iron oxide [16], IR820 [17], NaGdF_4_:Yb/Er [20], gold nanoparticles [21,25,26], copper hydroxide [22], black phosphorus [27]
	AMF	Ferrites [19,29,30], pure iron [31]
Photodynamic therapy	NIR	Zinc phthalocyanine [33], indocyanine green [34], gold nanoparticle [35], chlorine e6 [20,21,29,38,41,42]
Radiotherapy	Ionizing Radiation	Hafnium oxide [43,44,45], tungsten [46], bismuth [47]

## Abbreviations

AR	chimeric antigen receptor
ICD	immunogenic cell death
DAMPs	damage-associated molecular patterns
ER	endoplasmic reticulum
Hsp70	heat shock protein 70
NK	natural killer
DC	dendritic cell
HMGB1	high mobility group box 1
TLRs	toll-like receptors
mtDNA	mitochondrial DNA
CpG	cytosine-guanine
IFNs	interferons
TNFs	tumor necrosis factors
ILs	interleukins
NIR	near-infrared
PTT	photothermal therapy
IDO	indoleamine 2,3-dioxygenase
PDT	photodynamic therapy
MMP2	matrix metalloproteinase 2
ROS	reactive oxygen species
PEG	polyethylene glycol
